# Characterisation of Korean rice wine (*makgeolli*) prepared by different processing methods

**DOI:** 10.1016/j.crfs.2022.100420

**Published:** 2022-12-27

**Authors:** Barry Wong, Kevin Muchangi, Edward Quach, Tony Chen, Adrian Owens, Don Otter, Megan Phillips, Rothman Kam

**Affiliations:** a Department of Food Science and Microbiology, Auckland University of Technology, New Zealand; b Department of Marketing, Auckland University of Technology, New Zealand

**Keywords:** Korean rice wine, *Nuruk*, *Makgeolli* processing, AccQ·Tag™ amino acid analysis, Methyl-chloroformate derivatisation, Polarised project mapping (PPM)

## Abstract

Four methods of preparing *makgeolli,* a traditional Korean turbid rice wine, were reported in this study. The four processing routes include single-stage simultaneous saccharification and fermentation of glutinous rice with *nuruk* – a Korean starter culture (1SF-N), single-stage fermentation with *nuruk* and yeast (1SF-YN), two-stage fermentation (2SF) and three-stage fermentation (3SF). Chemical analysis was used to determine how the different processing routes could affect the rice wine's properties in terms of alcohol content, pH, colour, mineral content, proximate composition, antioxidant activity, total phenolic content, sugar, free amino acid, and organic acid profile. Sensory analysis using polarised projective mapping (PPM) and 62 participants found that sweetness is the most desirable attribute for *makgeolli* among New Zealand consumers with sourness and bitterness as less desirable. The 2SF *makgeolli* sample had the highest concentration of glucose (8.2 mg/mL) and maltose (107 mg/mL) and in the PPM experiment was the most preferred out of the four processing methods. The 1SF-N *makgeolli* sample had the highest alcohol (13% ABV), crude protein (4.9%), antioxidant activity, total phenolic (621 mg GAE/L) and free amino acids content, however, it was the least overall liked *makgeolli* sample. Overall, the novelty of this research includes formulating a traditional Korean turbid rice wine in a Western country environment and evaluating consumer perception of *makgeolli* beyond the normal clientele in South Korea. From these results it is suggested that the properties of *makgeolli* can be manipulated via processing to suit the brewer's sensory needs that best fits the consumer market.

## Highlights

• Four different methods of producing Korean rice wine (*makgeolli)* were identified.
• *Makgeolli* samples were tested for their physicochemical properties.
• Polarised projective mapping was conducted on *makgeolli* outside of the laboratory setting.
• Sweetness is the most desirable attribute for *makgeolli* among New Zealand consumers.
• Sweetness, sourness, and bitterness were the main drivers to determine participants' liking of *makgeolli.*

## Graphical abstract

**Figure:**
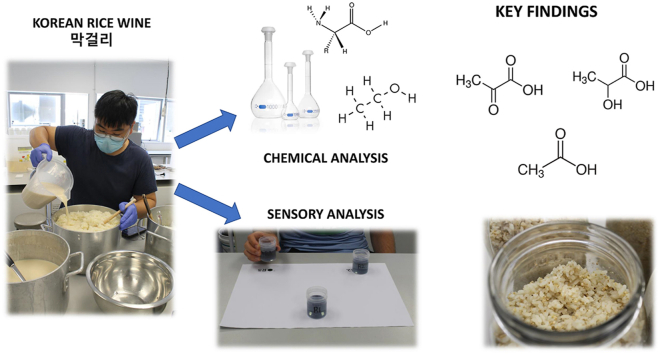

## 1 Introduction

*Makgeolli*, also known as Korean turbid rice wine, is a traditional Korean alcoholic beverage made from fermentation of glutinous rice with a starter culture called *nuruk* (Kim et al., 2015; Lee et al., 2015; Son et al., 2018). The beverage can be described as a whitish to yellowish opaque liquid with a sweet fruity aroma, slightly bitter, alcoholic, and sour taste. Most commercial *makgeollis* have an alcohol content of about 6–8% by volume and a pH between 3.6 and 4.4 (Jung et al., 2014). *Nuruk* is a traditional Korean rice wine starter culture made by grinding wheat, barley and rice followed by the addition of water to proliferate microorganism growth. The microbes in the *nuruk* depend on the grain source and the fermentation environment, hence the microbial community can vary significantly between products (Kwon and Sohn, 2012). However, the main microbes essential for the fermentation of *makgeolli* are *Saccharomyces cerevisiae*, *Aspergillus* spp, *Lactobacillus* spp, *Rhizopus* spp, and *Penicillium* spp. The α-amylase, β-amylase, glucoamylase enzymes produced by *Aspergillus* spp and *Rhizopus* spp are responsible for the saccharification of rice starch. *Saccharomyces cerevisiae* are responsible for converting the sugars into alcohol and fruity esters while the *Lactobacillus* spp creates a tarty flavour in *makgeolli* (Lee et al., 2007).

Previous studies have mainly focused on the metabolites, volatiles, and microbial profiles from a single stage fermented (1SF) *makgeolli* (Jeon et al., 2010; Kim et al., 2013; Lee et al., 2013). This is the most common *makgeolli* fermentation process and involves washing and soaking of the glutinous rice, followed by draining and steaming. Next, the rice is cooled to 25 °C and mixed with water, *nuruk* and additional yeast (*S. cerevisiae*) to initiate the fermentation process. After seven days of fermentation at room temperature, the mixture is filtered and bottled to give the final *makgeolli* product. There are other *makgeolli* processing routes that have been reported such as a two-stage (2SF) (Kang et al., 2014a; Kim et al., 2015) and a three-stage fermented (3SF) *makgeolli (**Nile, 2015**)*. The reasons for using multiple stage fermentations however were not stated and are not immediately obvious. The most apparent advantages of 1SF over 2SF and 3SF are the lower production time and the lower energy input. As mentioned earlier, 1SF *makgeolli* only requires seven days of fermentation but 2SF and 3SF *makgeolli* processes would require 10 and 12 days of fermentation respectively and require more processing steps. In summary, the 2SF and 3SF *makgeolli* products have been studied to a much lesser extent in terms of their chemical, nutritional, physicochemical, and sensory properties. Therefore, this study will investigate whether 2SF and 3SF may offer advantages over the 1SF process when producing *makgeolli*. As *makgeolli* becomes a more globally accepted beverage, it is of interest to determine the sensory preferences of other non-Korean consumers. A study of United States consumers found that a sweeter Korean rice wine was preferred (Kwak et al., 2015).

This work is divided into two parts. The first part aims at filling the knowledge gap by comparing and investigating the antioxidant activity, total phenolic content, amino acid profile, sugar profile, organic acid profile, mineral content, and the major physicochemical properties of *makgeolli* products made using 1SF, 2SF and 3SF processing routes. The second part involves the use of the polarised projective mapping (PPM) technique for sensory evaluation to show how New Zealand consumers in particular perceive *makgeolli*, as little is known about consumers’ perception of this beverage outside of Korea.

This mixed method approach is novel to food science because most food studies focus on either food chemistry or sensory science. When the two different fields of food science are combined, sensory acceptance tests are typically used to establish the overall liking of the food product. These tests do not necessarily further improve our understanding of how food products are perceived unless other sensory science tests such as discrimination or descriptive testing are utilised.

The significance of this study will allow the rice wine industry and brewers to select the most suitable processing route to produce a *makgeolli* with desirable attributes for non-Koreans and it also contributes to the evaluation of a relatively new method in the field of food sensory research (Ares et al., 2013; De Saldamando et al., 2015; Horita et al., 2017; Wilson et al., 2018) by applying the technique to holistic evaluations and perceptions of *makgeolli*.

## 2 Materials and method

### 2.1 Materials

#### 2.1.1 Makgeolli materials

Glutinous rice (*Oryza sativa* var. *glutinosa*), short grain non-glutinous rice (*Oryza sativa* subsp. *japonica*) and wheat based-*nuruk* were sourced from Wang Ltd., Korea. Safale US-05 dry ale yeast was sourced from Fermentis, S.I. Lesaffre, France.

#### 2.1.2 Chemicals and reagents

Trolox, 2,4,6-Tris(2-pyridyl)-s-triazine (TPTZ), neocuproine, gallic acid, Folin-Ciocalteu reagent, methanol, pyruvic acid, malonic acid, fumaric acid, citric acid, dry acetonitrile, phenol, D4-alanine, amino acid standard, pyridine and multielement standard solution for microwave plasma atomic emission spectroscopy (MP-AES) were purchased from Sigma-Aldrich, US. Malic acid, sucrose, D(+)galactose anhydrous and D(+)maltose were sourced from British Drug Houses (BDH) Chemicals Limited, UK. Iron (III) chloride, succinic acid, ascorbic acid, and anhydrous sodium sulphate were bought from Scharlab, Spain. Petroleum ether (analytical grade, 60–80 °C), hydrochloric acid (37%), sulphuric acid (97%), ammonium acetate, ammonium heptamolybdate, nitric acid (68%), formic acid, glacial acetic acid, acetonitrile, boric acid, potassium sulphate, sodium bicarbonate, sodium hydroxide, sodium carbonate and copper(II) sulphate were purchased from Thermo Fisher Scientific, US. Monopotassium phosphate was purchased from Interchem, NZ. Copper(II) chloride was purchased from VWR International, US. Methyl chloroformate were obtained from Merck, US. Absolute ethanol was obtained from Pauling Industries Limited, New Zealand. Chloroform was bought from Acros Organics, Spain. D(+)glucose anhydrous was purchased from Biolab limited, Australia. Borate was sourced from Pure Science Ltd., New Zealand. AccQ·Tag™ reagent for amino acids analysis was purchased from Apollo Scientific Ltd., UK. All chemicals were reagent grade.

### 2.2 Preparation of makgeolli

Preparation of single-stage fermented (1SF-N) *makgeolli* with *nuruk* involved weighing 2 kg of glutinous rice, followed by washing until the water ran clear (Jeon et al., 2010). The rice was soaked for 3 h, then drained for 1 h before subjecting to steaming for 120 min. The rice was then cooled to 25 °C followed by mixing with 3 L of de-ionised (DI) water and 500 g of ground *nuruk.* The mixture was fermented at 25 °C for 7 days. The resulting *makgeolli* (around 2 L) was filtered, bottled, and stored in a fridge at 4 °C until further analysis. Samples are analysed within three days from production.

Single-stage fermented yeast and *nuruk* (1SF-YN) *makgeolli* was made by the method reported by Kim et al. (2013). The preparation was similar to 1SF-N *makgeolli* with some modifications. The modifications were 40 g of ground *nuruk* and 11 g of dry yeast were used. The purpose of the yeast was to substitute a significant portion of the *nuruk.*

Preparation of two-stage fermented (2SF) *makgeolli* involved the same washing and draining process as 1SF-YN but also included the addition of a fermentation base (Kang et al., 2014b). The base was prepared by milling 400 g of non-glutinous rice into a fine powder using a blender (Russell Hobbs, UK). The resulting powder was mixed with 1.25 L of DI water and cooked for 15 min and cooled to 25 °C, followed by addition of 200 g of ground *nuruk* and then left to ferment for 1 day at 25 °C. This was used as a fermentation base for the next stage. Two kilograms of glutinous rice were washed, drained, and steamed in the same fashion as preparing for 1SF. One litre of the fermentation base was mixed with 1.5 L of DI water and steamed glutinous rice followed by 7-day fermentation at 25 °C.

**Fig. 1:**
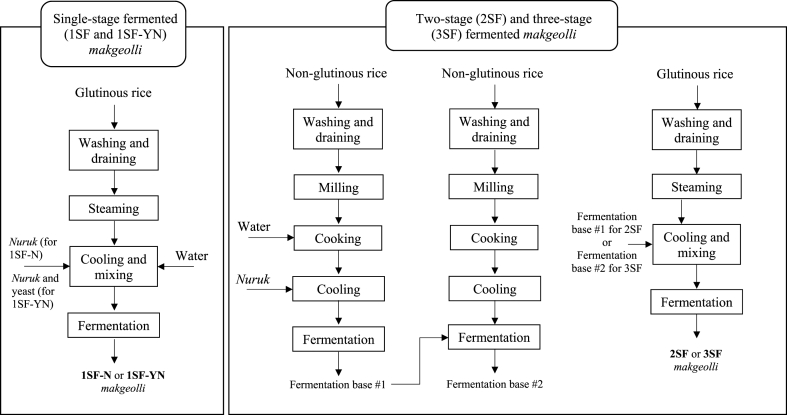
Process flow diagram showing four methods of making *makgeolli*.

Preparation of three-stage fermented (3SF) *makgeolli* followed the same procedure as 2SF except the fermentation base was fermented twice. For example, 1 L of the fermented non-glutinous base was added into another 400 g of washed, drained, milled and cooked non-glutinous rice. This was left to ferment without adding *nuruk* for 1 day at 25 °C and subsequently this second fermentation base was added to the steamed glutinous rice for the main fermentation. A summary of the preparation of these four styles of *makgeolli* are depicted in Fig. 1 and all the *makgeolli* samples were prepared in quadruplicate for analysis.

### 2.3 Proximate, alcohol, acetic acid, pH, minerals and colour analysis

Standard Association of Official Analytical Chemist (AOAC) methods were used to analyse for ash (method 942.05) (Thiex et al., 2019b), protein (method 954.01) (Bicsak, 2020) and lipid (method 920.39) (Thiex et al., 2019a) in the *makgeolli* samples. The AOAC method 925.09 for moisture content was not used because oven drying would caramelise the carbohydrates and give inconsistent results. Instead, the *makgeolli* samples were freeze-dried in a Christ® Alpha 2–4 LSC Plus freeze dryer (Germany) for 5 days to determine the moisture content. Total carbohydrate was determined using the sulphuric acid-phenol method described by Chow and Landhäusser (2004).

The concentration of ethanol and acetic acid were measured by GC-FID using a Shimadzu GC-2010 Plus gas chromatograph (Shimadzu, Japan) equipped with a split/splitless injector, a flame ionisation detector (FID) and fitted with an Agilent Durabond – Fatwax Ultra Inert column which has 30 m length x 0.25 mm i. d. x 0.25 μm film thickness (Agilent, USA). The alcohol concentration was reported in percentage alcohol by volume (ABV).

Colour determination was performed using the protocol of Tangsrianugul et al. (2019). The equipment used for analysis was a Hunter lab ColorFlex 45/0 V1.80 spectrophotometer (HunterLab, Virginia, USA). Colour values were given as CIE L* (light/dark), a* (+red/-green) and b* (+yellow/-blue).

Minerals analysis was done according to the principle of Poitevin (2019). After digesting with concentrated nitric acid in a microwave digester (Anton Paar Multiwave GO Plus, Austria) and dilution, the samples were analysed with an Agilent Technologies 4200 microwave plasma atomic emission spectrometer (USA).

The pH was measured using an Oakton pH 700 Bench Meter (Oakton Instruments, USA).

### 2.4 Antioxidant capacity and total phenolic content

Three different antioxidant assays were carried out on the *makgeolli* samples to check for false positives/negatives. All the *makgeolli* samples were diluted by a factor of 10 before analysis and all the antioxidant results were expressed in mg Trolox equivalent (TE) per L of *makgeolli* through comparison to a standard curve.

The *phosphomolydenum (phospho.) assay* was performed as outlined by (Ivanišová et al., 2016). One mL of diluted *makgeolli* sample was mixed with 2.8 mL KH_2_PO_4_ (0.1 M), 6 mL H_2_SO_4_ (1 M), 0.4 mL (NH_4_)_6_Mo_7_O_24_ (0.1 M) and 0.8 mL of distilled water in a glass vial. Once vortexed, the samples were incubated at 90 °C for 120 min. The samples were rapidly cooled in an ice water bath, then the absorbance measured at 700 nm.

The *ferric-ion reducing antioxidant power (FRAP) assay* involved the following procedure according to Benzie and Strain (1996). 0.1 mL of diluted *makgeolli* was mixed with 2 mL of FRAP reagent and 0.9 mL of distilled water and then left to react for 5 min at ambient temperature. The absorbance of the samples was measured at 593 nm.

The *cupric reducing antioxidant capacity (CUPRAC) assay* is described by Özyürek et al. (2011) with slight modifications. One mL of diluted *makgeolli* was added to 1 mL CuCl_2_ (0.01 M), 1 mL of NH_4_Ac (1 M), 1 mL of neocuproine (0.0075 M) and 0.1 mL of distilled water. The solution was left to react for 5 min at ambient temperature and its absorbance was measured at 450 nm.

The *Folin-Ciocalteu (FC) total phenolic assay* was carried out according to the method reported in Singleton et al. (1999) with slight modifications. Ten-fold diluted *makgeolli* with distilled water (1 mL) was mixed with 500 μL of FC reagent in a glass vial and left to react for 5 min at ambient temperature. Twenty percent (m/v) Na_2_CO_3_ (1.5 mL) was then added to the solution and vortexed for 10 s. The glass vial was left to react for 2 h in the dark. The absorbance was measured at 765 nm. Total phenolic content was expressed in mg gallic acid equivalents (GAE)/L *makgeolli*.

### 2.5 Sugar analysis by high performance liquid chromatography coupled with evaporative light scattering detector (HPLC-ELSD)

Glucose, galactose, and maltose were used as the calibration sugar standards. An adaptation of a procedure by Chessum et al. (2022), was used. Briefly, the 1SF-N, 1SF-YN and 3SF *makgeolli* samples were diluted tenfold using ultrapure water. For the 2SF *makgeolli* sample, a 25-fold dilution was performed. The diluted *makgeolli* samples (3 mL) and 60 μL of xylitol (internal standard) were mixed with 3 mL of chloroform to create a monophasic liquid. The solution was vortexed at medium speed for 30 s. The solution was then centrifuged at 1300×*g* for 10 min. The bottom chloroform part was discarded. This process was repeated two more times. One millilitre of the upper aqueous part was then drawn out and centrifuged at 1300×*g* at 4 °C for 10 min before HPLC analysis. A Shimadzu-10 A Auto Injector (Shimadzu Corporation, Japan) coupled to a Shimadzu LC-10ATVP pump were used for the liquid chromatography analysis. Separation of sugars was achieved using a Luna® Omega 3 μm SUGAR 100 A 250 × 4.6 mm LC Column (Phenomenex, USA). The mobile phase was 80% Acetonitrile – 20% Milli-Q water, isocratic dilution. Injection volume was 5 mL at a flow rate of 0.5 mL/min. An Agilent 385-ELSD detector (Agilent Technologies, USA) was used for the detection of compounds. Instrument grade nitrogen gas (BOC) controlled by an Agilent N2-14-K727 nitrogen gas generator was set at a flow rate of 1.2 mL/min. The evaporator temperature was 80 °C, and the nebuliser temperature was 50 °C.

### 2.6 Organic acids analysis by methyl chloroformate (MCF) derivatisation

The all standards and samples were frozen using liquid nitrogen before freeze-drying overnight in a freeze-dryer (Virtis SP Scientific Advantage Pro, USA). Derivatisation with methyl chloroformate (MCF) was done according to the protocol of Smart et al. (2010) with d4-alanine as the internal standard. An Agilent 7890 B gas chromatograph coupled to an Agilent 5975 B single quadrupole mass spectrometer (Agilent, USA) and a Gerstel multipurpose autosampler (Gerstel, Germany) were used for gas chromatography – mass spectrometry analysis. Briefly, 1 μL of each sample was injected into a 4 mm glass splitless inlet packed with deactivated glass wool. The inlet temperature was 290 °C; the column flow was 1.0 mL/min; and the initial average linear velocity was 35 cm/s. Purge flow was set at 25 mL/min, 1 min after injection. The column used was a fused silica DB-1701 30 m × 0.25 mm x 0.25 μm stationary phase (86% dimethylpolysiloxane, 14% cyanopropyl phenyl) Agilent column (Agilent technologies, USA). The carrier gas used was instrument grade (99.99%, BOC) helium gas. GC oven temperature was set as follows: The temperature was raised to 45 °C and held for 2 min, then increased at a rate of 9 ^°^C/min to 180 °C and held at this temperature for 5 min. The temperature was again increased by 40 ^°^C/min to 220 °C where it was held for 5 min. The temperature was increased again at a rate of 40 ^°^C/min to 240 °C and held for 11.5 min. The temperature was finally ramped up by 40 ^°^C/min to 280 °C and held for 10 min. The transfer line to the mass spectrometric detector was maintained at 250 °C, the source at 230 °C and the quadrupole at 150 °C. The detector was turned on 5.5 min into the run. The detector was run in positive-ion, electron-impact ionisation mode, at 70eV electron energy. MassHunter software (version b.07.01 sp1; build 7.1.524.1), was used to interpret the data. Identification of compounds was performed by comparing the results to the National Institute of Spectral library (NIST, version 2.2; build Jun 10, 2014).

### 2.7 Amino acid analysis by AccQ·Tag™ method

The AccQ·Tag™ method for detecting amino acids, calibration standards and sample preparation were performed according to Salazar et al. (2012). Amino acids were analysed in an Agilent 1260 Infinity Quaternary LC System (USA). The system consisted of the following components: 1260 quaternary pump, 1260 infinity automatic liquid sampler, 1260 infinity thermostatted column compartment, and 1260 infinity diode array detector, connected to a 6420 triple quadrupole liquid chromatograph-mass spectrometry detector system with electrospray ionisation source. Separation of amino acids was achieved with a Kinetex® 1.7 μm Evo C18 100 A 2.1 × 150mm liquid chromatograph column. The stationary phase was C18 with trimethylsilyl end-capping while the solid phase was a core-shell organo-silica with ethane cross-linking. The column was operated at 25 °C. Mobile phase A was 0.1% formic acid in MilliQ water. Mobile phase B was 0.1% formic acid in acetonitrile. The flow rate was set at 0.225 mL/min. The mass spectrometer ionisation source conditions were as follows: capillary voltage of 4 kV, drying gas temperature of 300 °C, drying gas flow of 10 L/min and nebuliser pressure of 30 psi. The positive ion mode was performed with multiple reaction monitoring (MRM) for quantitative analysis. MassHunter software, (version b.09.00/build 9.0.647.0) was used for analysis. Identification of compounds was done by comparing results to the National Institute of Spectral library (NIST, version 2.2; build Jun 10, 2014).

### 2.8 Sensory evaluation of makgeolli using polarise projective mapping (PPM)

#### 2.8.1 Participant recruitment and sociodemographic

The sensory experiment was carried out in Auckland, New Zealand and participants were recruited by friends and staff members of AUT University. Due to the ongoing situation of COVID-19 in Auckland, New Zealand between late 2021 and early 2022, the sensory experiment shifted from a laboratory setting to a take-home test in form of sensory test kits (*refer to* Supplementary Information SI.1*)*. This method was used to minimise the potential spread of COVID-19 among the participants and to adhere to health and safety policies. The criteria that participants must fulfilled before partaking in the sensory experiment included reading through the information sheet, being over the legal age of 18 for consuming alcohol, not suffering from food allergies such as wheat, rice, and alcohol, not operating heavy machinery or driving within 2 h of completing the experiment, and not being pregnant or trying to conceive a child.

Sixty-two participants took part in the sensory experiment with an even split between males and females. For ethnicity, 40.0% of the participants were Chinese, followed by 20.0% Koreans, 12.9% Caucasians and 8.1% were categorized as other including Iranian, Latin American, Middle Eastern, Pacific Islander and Vietnamese. 33.9% of the participants were between the ages of 21–29 years, followed by 29.03% aged between 30 and 39 years, 14.5% aged 40–49 years, and 25.8% were over the age of 50 years.

#### 2.8.2 Sensory materials

The test kit included a set of seven 50 mL solutions, a questionnaire, instructions with photos, a consent form, a pen, and a large plastic bag 27 cm × 33 cm (Armada, New Zealand). Sensory test kits were sent out to the participants and collected by the researcher.

The seven solutions consisted of three pole solutions including R1 (sucrose 10 g/L – sweet), R2 (citric acid 2 g/L – sour), R3 (quinine 6 mg/L – bitter) and the four *makgeolli* samples (1SF-N, 1SF-YN, 2SF, and 3SF). Fifty milliliters of pole solutions and samples were placed in sealed 70 mL sterile leakproof plastic containers (Citotest, China). The pole solutions were labelled with R1, R2, and R3 while the *makgeolli* samples were labelled with three-randomised digits. The test kits were stored at 4°C and the participants head to conduct the sensory test within three days after receiving the package.

#### 2.8.3 Polarised projective mapping (PPM) sensory procedures and questionnaire

The sensory experiment included four components. Firstly, participants were tasked to complete the PPM experiment and then followed this by indicating their degree of liking on a 100 mm bi-polar line scale of the poles and the *makgeolli* samples. Secondly, participants were then asked about their subjective and objective knowledge of wine. Lastly, sociodemographic data including gender, age, ethnicity, and frequency of consuming alcohol were collected.

The three-pole solutions were placed 25 cm apart in a triangle formation on an A3 piece of paper (*refer to Supplementary Information SI.2* for an example). Using the bottom left corner (landscape position) of the A3 piece of paper as a reference point, the coordinates of the three-pole solutions are placed on three fixed points either X1 = 25.6 cm and Y1 = 21.0 cm, X2 = 4.0 cm and Y2 = 8.6 cm, and X3 = 4.0 cm and Y3 = 33.5 cm. The triangle formation enabled the participants to place the four different *makgeolli* samples within the triangle space.

Participants were asked to sample and remember the taste, flavour, and smell of the pole solutions (e.g., R1, R2, and R3)and then were asked to consume the *makgeolli* samples in no specific order and arrange the *makgeolli* samples on the A3 paper using the three poles as a guide. Participants were instructed to arrange the *makgeolli* samples and poles closer to each other if the subjects believe the sensory attributes were similar, while *makgeolli* samples and poles that were not similar were placed further apart. On the instruction sheet and questionnaire, participants were told that there were no right or wrong answers, and the placement of the *makgeolli* was based on their sensory perception and to use their own criteria for the *makgeolli* arrangement.

### 2.9 Statistical analysis

#### 2.9.1 Statistical analysis on chemical data

Statistical analysis was performed using R (version 3.6.2; 2019-12-12). The Shapiro test was carried out to check for normality of the data; p-value >0.05 demonstrated that data was normally distributed. Correlation of normally distributed data was performed using the Pearson correlation coefficient. Kendall correlation coefficient was used for data not normally distributed. A p-value <0.05 meant the two variables under investigation were statistically significantly correlated. Only the data sets meeting these criteria (p-value <0.05) were reported in this study. One way analysis of variance (ANOVA) and Tukey honest significant difference (Tukey HSD) tests were also performed to check for significant differences (p-value <0.05) between the sample mean values.

#### 2.9.2 Statistical analysis on sensory data

The statistical analysis of the PPM experiment and degree of liking was separated into three parts: overall, male and female. Using the left bottom corner as a reference, all seven solutions including poles and *makgeollis* were manually measured for their X and Y coordinates. The coordinates of each subject were then recorded on a table with two columns for each pole and sample, representing X and Y coordinates on the excel spreadsheet. Once all the coordinates are established, multi-factor analysis (MFA) was used to analyse the PPM data.

One-way ANOVA was used to establish whether there were statistical differences in hedonic liking between the poles and *makgeolli* samples for overall, male, and female. Two-way ANOVA was used to see whether the hedonic liking of poles and *makgeollis* were affected by gender. Results were considered statistically significant on liking when the associated p-value was <0.05. R software version was used for statistical analysis, R version 4.1.3. FactoMineR and factoextra packages were used for MFA, and Agricola packages were used for the Tukey HSD posthoc test in both one-way and two-way ANOVA.

## 3 Results and discussion

### 3.1 Proximate analysis and physicochemical properties of makgeolli

The proximate composition, alcohol, pH, colour, and mineral content of the *makgeolli* samples are shown in Table 1. Reports on proximate composition of *makgeolli* are scarce in the literature with only one study by Park et al. (2015). Park et al. (2015) reported that their *makgeolli* products in general have an average of 92.7% moisture, 3.8–4.38% total carbohydrate and 2.5–3.0% total protein. These values were different to the results from this study due to the different rice used for making their *makgeolli* samples. Park et al. (2015) used two non-glutinous *Japonica* rice cultivars *Seolgaeng* and *Sindongjin* whereas the *makgeolli* samples from this study were produced using glutinous rice. Therefore, it is expected that the moisture content would be lower at 78–84% and the carbohydrate would be higher at 12–20% although the initial water to grain ratio for making these *makgeolli* samples was roughly the same as used by Park et al. (2015).

**Table 1:** Proximate composition, alcohol content, pH, colour, mineral content, antioxidant activity and total phenolic content in *makgeolli* made with four different processing routes.

Makgeolli sample	Proximate composition (% wet basis)					Alcohol (%ABV)	pH	Colour		
	Moisture	Total carbohydrate	Total protein	Ash	Lipid			L*	a*	b*
1SF-N	82.37 ± 1.22^a^	12.55 ± 1.43^a^	4.89 ± 0.17^a^	0.29 ± 0.05^a^	0.06 ± 0.05^a^	13.0 ± 0.5^a^	3.73 ± 0.03^a^	61.3 ± 0.4^a^	0.495 ± 0.1^a^	10.91 ± 0.1^a^
1SF-YN	84.31 ± 1.51 ^a^	14.17 ± 1.34^a^	1.35 ± 0.34^b^	0.06 ± 0.06^b^	0.11 ± 0.04^a^	4.5 ± 0.4^b^	3.42 ± 0.05^b^	42.4 ± 0.1^b^	−1.92 ± 0.1^b^	−1.06 ± 0.4^b^
2SF	78.10 ± 2.10 ^b^	20.43 ± 2.34^b^	1.42 ± 0.25^b^	0.09 ± 0.04^b^	0.06 ± 0.07^a^	7.8 ± 0.4^c^	3.62 ± 0.03^c^	56.9 ± 2.0^c^	−1.29 ± 0.2^c^	6.35 ± 0.5^c^
3SF	84.47 ± 1.78 ^a^	13.55 ± 1.61^a^	1.74 ± 0.41^b^	0.08 ± 0.07^b^	0.15 ± 0.06^a^	12.6 ± 0.5^d^	4.14 ± 0.12 ^d^	58.4 ± 1.4^c^	−1.28 ± 0.2^c^	3.99 ± 0.2^d^

Makgeolli sample	Mineral content in mg/L									
	P	Zn	Si	Ca	Cu	K	Mg	Mn	Fe	Na
1SF-N	10.61 ± 0.44^a^	0.43 ± 0.16^a^	1.07 ± 0.28^a^	2.47 ± 1.10^a^	0.27 ± 0.15^a^	11.61 ± 1.84^a^	2.45 ± 0.37^a^	0.56 ± 0.01^a^	1.24 ± 0.52^a^	1.28 ± 0.18^a^
1SF-YN	19.61 ± 3.98^b^	0.31 ± 0.07^b^	0.94 ± 0.39^b^	2.33 ± 0.55^b^	0.18 ± 0.04^b^	5.24 ± 0.78^b^	0.75 ± 0.04^b^	0.49 ± 0.02^b^	0.76 ± 0.10^b^	2.00 ± 0.71^b^
2SF	21.03 ± 4.54^c^	0.27 ± 0.01^c^	0.23 ± 0.19^c^	1.80 ± 0.16^c^	0.18 ± 0.03^b^	7.30 ± 1.22^c^	1.45 ± 0.13^c^	0.50 ± 0.02^c^	0.55 ± 0.08^c^	1.49 ± 0.31^c^
3SF	26.83 ± 2.13^d^	0.31 ± 0.01^d^	0.11 ± 0.14^d^	2.22 ± 0.45^c^	0.13 ± 0.05^d^	7.17 ± 1.67^c^	1.22 ± 0.23^c^	0.52 ± 0.01^c^	0.60 ± 0.17^c^	1.42 ± 0.18^c^

Makgeolli sample	Antioxidant Activity (g TE/L)			Total Phenolic Content (mg GAE/L)
	Phospho	FRAP	CUPRAC	Folin-Ciocalteu
1SF-N	3.99 ± 0.29^a^	0.65 ± 0.15^a^	0.48 ± 0.02^a^	621 ± 9^a^
1SF-YN	2.81 ± 0.25^b^	0.27 ± 0.02^b^	0.17 ± 0.09^b^	307 ± 8^b^
2SF	2.13 ± 0.20^c^	0.26 ± 0.01^b^	0.24 ± 0.02^b^	397 ± 10^c^
3SF	3.01 ± 0.22^b^	0.24 ± 0.03^b^	0.22 ± 0.01^b^	318 ± 19^d^

Results are expressed as the mean ± standard deviation (n = 4).Values with different a – d superscript letters in the same column differ significantly (p < 0.05) as determined by Tukey test. TE = Trolox equivalent. GAE = Gallic acid equivalent.

The 1SF-N *makgeolli* had the highest protein content because of the comparatively high concentration of *nuruk* used for the fermentation process. Kang et al. (2016) conducted a proximate analysis on *nuruk* and found that the crude protein in wheat-based *nuruk* can vary between 12% and 14%. The pH values of the *makgeolli* samples in this study were within the expected range of 3.4–4.5 as reported by other research groups (Jung et al., 2014; Lee et al., 2013; Park et al., 2015).

The alcohol content for the 1SF-N *makgeolli* was the highest at 13.0% ABV and was assumed to be due to the large amount of *nuruk* used for the simultaneous saccharification and fermentation (SSF) step. This alcohol level is similar to alcohol levels reported by Park et al. (2015) (14–18% alcohol) but higher than 6–7% levels reported for *makgeolli* by Kim et al. (2013). As the *nuruk* contains fungi that produces amyloytic enzymes for the saccharification process and yeasts that facilitate alcohol production (Bal et al., 2014), the 1SF-N fermentation process is expected to be more efficient in generating alcohol. The 3SF *makgeolli* also has a high alcohol content (12.6%) because of the double pre-fermentation step to produce a starter base that may also contain high numbers of microbes for the main fermentation. The 1SF-YN *makgeolli* has the lowest alcohol content at 4.52%. This result was expected even though this sample had added yeast it has the lowest amount of *nuruk* used during the SSF step..

Phosphorus was observed to have the highest concentration in the four types of *makgeolli* tested. Calcium, potassium, sodium, and magnesium were observed to be in moderate concentrations. The lowest concentration of minerals observed in the four *makgeolli* samples were zinc, silicon, copper, and manganese (Table 1). All these minerals are important for the microbial activity (Freire et al., 2017) during fermentation of *makgeolli* samples.

In terms of colour 1SF-N *makgeolli* was observed to be the darkest (L* = 61.3), reddest (a* = 0.495) and yellowest (b* = 10.91) among the four *makgeolli* samples. This is due to the high tannic content in the *nuruk* (Jeon et al., 2010) resulting in a strong brownish hue in the *makgeolli*. The redness (a* values) were low in the other *makgeolli* samples (<-1.28), whereas the yellowness (b* values) were in the range between −1.06 and 6.35.

The total phenolic content (TPC) of the *makgeolli* samples from this study were in general higher than those reported in commercially available *makgeolli* samples in Korea. Bae et al. (2010) reported that the TPC in commercial *makgeolli* products ranged between 100 and 250 mg GAE/L. The TPC of the *makgeolli* samples from this study ranged between 300 and 400 mg GAE/L with the exception of 1SF-N which had 621 mg GAE/L. This elevated TPC value was because of 1SF-N sample had the highest amount of *nuruk* incorporated into the fermentation process and is similar to the TPC values of 600–1400 mg GAE/L reported by Lee (2020). As mentioned earlier, *nuruk* is a starter culture predominately made of ground wheat and barley which are known to contain sizable amounts of phenolic compounds such as ferulic acid, syringic acid, p-hydroxybenzoic acid, p-coumaric acid and hydroxycinnamic acids (Hernández et al., 2011; Quinde-Axtell and Baik, 2006). Interestingly, the TPC of these rice wines were much lower than red grape wines but higher than white grape wines which have been reported to have TPC values of 1200–2500 mg GAE/L and 150–300 mg GAE/L respectively (De Beer et al., 2003; Lingua et al., 2016).

All the *makgeolli* samples exhibited some antioxidant activity with 1SF-N giving the highest activity. It is known that dietary phenolic compounds are effective antioxidants (Rice-Evans et al., 1997). Therefore, the high phenolic content in *nuruk* is presumed to be responsible for the high antioxidant activity in the 1SF-N sample. The phospho. antioxidant assay resulted in the highest antioxidant activity values of the three assays used in this study. This was not surprising as the three assays all measure different aspects of antioxidant activity. Also, the phospho. assay can overestimate antioxidant activity if there are other non-phenolic reducing agents such as reducing sugars and certain amino acids present in the sample (Munteanu and Apetrei, 2021). For the FRAP and CUPRAC assays, there were no significant differences in antioxidant activity among the 1SF-YN, 2SF and 3SF samples. The results from the FRAP assay in this study were comparable with reported literature values (Lee et al., 2022). However, since this is the first time the CUPRAC assay has been applied to *makgeolli,* any comparison to reported literature is difficult. In general, the antioxidant activity from the three essays showed similar a pattern to the TPC values, this is in agreement with Lee (2020). Interestingly, the antioxidant activity of *makgeolli* (0.24–0.65 g TE/L) were much lower than red wines (1.72–3.8 g TE/L) using FRAP assay as comparison (Kondrashov et al., 2009).

### 3.2 Sugars in makgeolli

Maltose was the most abundant sugar detected in the *makgeolli* samples from this study (Table 2), except in 1SF-N where it was not detected. This was in agreement with the observations of Son et al. (2018). The glucose concentration was, however, found to be low in all the *makgeolli* samples relative to maltose. The 2SF *makgeolli* sample was observed to contain the highest concentrations of both maltose and glucose among the tested *makgeolli* samples. This agrees with the high total carbohydrate in 2SF *makgeolli* as shown in Table 1 and similar results were reported by Lee et al. (2013) who found glucose, maltose and trace amounts of fructose in their samples. The use of the first fermentation base as a starter culture to ferment the main glutinous rice may have created an ideal conditions for the hydrolysis and saccharification of the amylopectin which accounts for 99.7% of the starch in glutinous rice (Guo et al., 2015). Also, this may be the reason for the moderate alcohol content and high glucose and maltose content in 2SF *makgeolli* as the yeast did not have enough time to convert the sugars to alcohol.

**Table 2:** Sugar content in mg/mL in the four types of *makgeolli* samples.

Sample	Glucose	Galactose	Maltose
1SF-N	5.13 ± 0.99^a^	0.38 ± 0.08^a^	ND
1SF-YN	4.43 ± 0.28^b^	0.14 ± 0.01^b^	67.70 ± 12.14^a^
2SF	8.24 ± 1.77^c^	0.32 ± 0.06^c^	107.93 ± 13.53^b^
3SF	6.10 ± 0.84^d^	0.25 ± 0.01^d^	11.33 ± 0.90^c^

Results are expressed as the mean ± standard deviation (n = 4). Values with different a – d superscript letters in the same column differ significantly (p < 0.05) as determined by Tukey test. ND = not detected.

### 3.3 Organic acids in makgeolli

Pyruvic acid was detected in the highest amounts in the 2SF *makgeolli* followed by the 1SF-YN *makgeolli*. However, it was detected in relatively low concentrations in the 3SF and 1SF-N *makgeolli* (Table 3). Fumaric acid was not detected in any of the *makgeolli* samples which corresponded to the findings of Seo et al. (2016). It is known that pyruvic, citric and fumaric acid are intermediate products of glycolysis and the citric acid cycle (Son et al., 2018). In particular, pyruvic acid supplies energy to microbial cells through the citric acid cycle in aerobic environments. However, in an anaerobic environment such as an alcoholic fermentation, the pyruvic acid is converted to ethanol (Yu et al., 2015). Since pyruvic acid is not the end-product of fermentation, the presence of high levels of pyruvic acid in the *makgeolli* samples would depict an incomplete glycolytic and citric acid cycle in the yeast cells. Therefore, it can be inferred that the fermentation process in the 2SF *makgeolli* and 1SF-YN *makgeolli* samples was incomplete. The 1SF-N and 3SF *makgeolli* samples had low levels of pyruvic acid, indicating a more complete fermentation process, andhence the higher alcohol content.

**Table 3:** Organic acids in g/L in the four types of *makgeolli* samples.

Sample	Pyruvic	Malonic	Succinic	Citric	Malic	Tartaric	Lactic	Acetic	Fumaric
1SF-N	7.37 ± 0.42^a^	0.02 ± 0.01^a^	0.47 ± 0.03^a^	0.05 ± 0.01^a^	0.05 ± 0.01^a^	0.06 ± 0.01^a^	8.79 ± 0.15^a^	0.72 ± 0.05^a^	ND
1SF-YN	15.61 ± 0.99^b^	0.02 ± 0.01^a^	0.07 ± 0.01^b^	0.01 ± 0.01^a^	0.02 ± 0.01^b^	0.004 ± 0.001^b^	6.37 ± 0.32^b^	2.23 ± 0.29^b^	ND
2SF	22.58 ± 1.60^c^	0.02 ± 0.01^a^	0.12 ± 0.01^c^	0.02 ± 0.01^a^	0.02 ± 0.01^b^	0.019 ± 0.001^c^	11.28 ± 0.08^c^	1.79 ± 0.37^c^	ND
3SF	1.13 ± 0.06^d^	0.02 ± 0.01^a^	0.47 ± 0.02^a^	0.02 ± 0.01^a^	0.03 ± 0.01^c^	0.005 ± 0.001^d^	1.55 ± 0.08^d^	0.33 ± 0.04^d^	ND

Results are expressed as the mean ± standard deviation (n = 4). Values with different a – d superscript letters in the same column, differ significantly (p < 0.05) as determined by Tukey test. ND = not detected.

Malic and citric acid were made by *S. cerevisiae* from glucose during fermentation. Malic acid is generated through the oxaloacetate or fumarate pathways. Citric acid is generated during conversion of sugar to alcohol by *S. cerevisiae* or during malolactic fermentation by lactic acid bacteria (Seo et al., 2016). In this study, concentrations of these two organic acids were highest in the 1SF-N *makgeolli* sample. This could therefore be indicative of the higher presence of *S. cerevisiae* from the *nuruk*. However, the concentrations of malic acid and citric acid were very low and did not vary significantly in all the other types of *makgeolli*.

Succinic acid, lactic acid and acetic acid were also produced by *S. cerevisiae* during the fermentation process (Seo et al., 2016; Son et al., 2018; Yu et al., 2015). Therefore, these organic acids could possibly be used as indicators of the efficiency of yeast metabolism. It was also discovered that succinic acid had a positive correlation to ethanol content (Seo et al., 2016). In this study, succinic acid concentration was highest in both the 1SF-N and 3SF *makgeolli* which also had the highest alcohol content.

### 3.4 Amino acids in makgeolli

Bacterial and fungal proteases from *nuruk* can break down proteins in rice and wheat into free amino acids (Seo et al., 2016). This may explain the high free amino acid content in all *makgeolli* samples (Table 4). Phenylalanine, glutamic acid, proline, and isoleucine were detected in high concentration whereas cysteine, citrulline, and ethanol amine were very low in the four *makgeolli* samples. This observation was in accord with a report by Kang et al. (2014b). The 1SF-N *makgeolli* sample contained the highest amount of total free amino acids which corresponded to the highest crude protein content due to amount of *nuruk* used. Conversely, 1SF-YN *makgeolli* had the least concentration of total amino acids and subsequently, a low crude protein content. Although the essential and non-essential free amino acids have little to no taste (Solms, 1969), understanding the free amino acid profile of *makgeolli* is very important because the amino acids can influence the development of the aroma and taste profile of the final beverage. For example, essential amino acids can be directly assimilated by yeast under the Ehrlich pathway where they are converted into aromatic and volatile compounds such as aldehydes, fusel alcohols and fusel acids (Hazelwood et al., 2008; Kang et al., 2014b; Son et al., 2018). These compounds have significant impact on the flavour of the final *makgeolli* (Kang et al., 2014a).

**Table 4:** Free amino acid concentration (mg/L) in the four types of *makgeolli* samples.

Makgeolli sample	Essential amino acids								
	Histidine	Leucine	Isoleucine	Lysine	Methionine	Phenylalanine	Threonine	Tryptophan	Valine
1SF-N	57.3 ± 3.9^a^	108.2 ± 12.4^a^	271.8 ± 31.5^a^	124.9 ± 4.8^a^	26.9 ± 4.5^a^	222.0 ± 13.1^a^	59.2 ± 4.8^a^	54.4 ± 3.4^a^	199.1 ± 13.8^a^
1SF-YN	1.9 ± 0.2^b^	9.4 ± 1.5^b^	33.2 ± 2.4^b^	11.7 ± 0.3^b^	4.0 ± 0.9^b^	51.0 ± 2.7^b^	6.1 ± 0.8^b^	15.2 ± 3.7^b^	31.3 ± 1.8^b^
2SF	23.0 ± 2.5^c^	55.4 ± 8.7^c^	136.2 ± 17.3^c^	82.8 ± 7.5^c^	9.8 ± 5.7^c^	126.5 ± 15.3^c^	27.8 ± 3.7^c^	24.2 ± 6.6^c^	98.7 ± 14.8^c^
3SF	46.9 ± 1.9^d^	49.0 ± 1.6^d^	117.7 ± 4.3^d^	105.6 ± 9.2^d^	1.7 ± 0.2^d^	106.2 ± 7.3^d^	42.7 ± 1.5^d^	17.4 ± 1.7^d^	87.1 ± 7.9^d^

Makgeolli sample				
Non-essential amino acids	1SF-N	1SF-YN	2SF	3SF
Alanine	385.8 ± 18.1^a^	49.5 ± 2.0^b^	178.0 ± 25.9^c^	321.5 ± 21.9^d^
Arginine	202.3 ± 17.8^a^	36.0 ± 2.0^b^	45.9 ± 3.9^c^	94.7 ± 19.6^d^
Aspartic acid	108.5 ± 10.1^a^	12.8 ± 0.8^b^	76.3 ± 11.6^c^	60.1 ± 2.1^d^
Citrulline	7.9 ± 0.7^a^	2.9 ± 0.4^b^	6.4 ± 0.3^c^	2.3 ± 0.2^d^
Cysteine	3.6 ± 0.2^a^	ND	0.6 ± 0.0^b^	0.2 ± 0.0^c^
Ethanol amine	7.5 ± 0.5^a^	2.0 ± 0.1^b^	3.9 ± 0.2^c^	6.6 ± 0.5^d^
Glutamic acid	334.3 ± 41.0^a^	61.4 ± 2.6^b^	164.9 ± 22.2^c^	92.6 ± 11.5^d^
Glycine	139.7 ± 10.2^a^	20.7 ± 1.2^b^	59.7 ± 11.5^c^	73.3 ± 3.8^d^
Proline	475.0 ± 37.6^a^	63.4 ± 4.4^b^	193.8 ± 18.8^c^	158.1 ± 12.9^d^
Hydroxy-Proline	17.7 ± 1.8^a^	1.9 ± 0.4^b^	4.2 ± 0.1^c^	4.3 ± 0.6^c^
Ornithine	84.4 ± 5.7^a^	1.9 ± 0.3^b^	119.6 ± 10.6^c^	91.4 ± 9.8^d^
Serine	93.5 ± 2.6^a^	10.4 ± 0.3^b^	49.6 ± 8.8^c^	72.0 ± 2.6^d^
Tyrosine	211.1 ± 19.8^a^	29.9 ± 3.9^b^	92.0 ± 5.8^c^	112.0 ± 7.1^d^
Total amino acids	3195.1 ± 77.3 ^a^	456.6 ± 9.3 ^b^	1579.3 ± 54.7 ^c^	1663.4 ± 39.6 ^d^

Results are expressed as the mean ± standard deviation (n = 4). Values with different a – d superscript letters in the same column, differ significantly (p < 0.05) as determined by Tukey test. ND = not detected.

In general, the non-essential amino acids were detected in high concentrations relative to the essential amino acids. Similar findings have been reported by Yu et al. (2015). Non-essential amino acids such as glycine, alanine, and serine were also assimilated by yeast. However, they could not be metabolised through the Ehrlich pathway, instead, they were converted to pyruvate. This could be further broken down into esters via the anabolic pathway (Pires et al., 2014). In the current study, the 1SF-YN *makgeolli* was observed to contain the lowest concentrations of the non-essential amino acids whereas 1SF-N *makgeolli* had the highest concentrations of the non-essential amino acids.

### 3.5 Polarised projective mapping sensory results on *makgeolli*

**Fig. 2:**
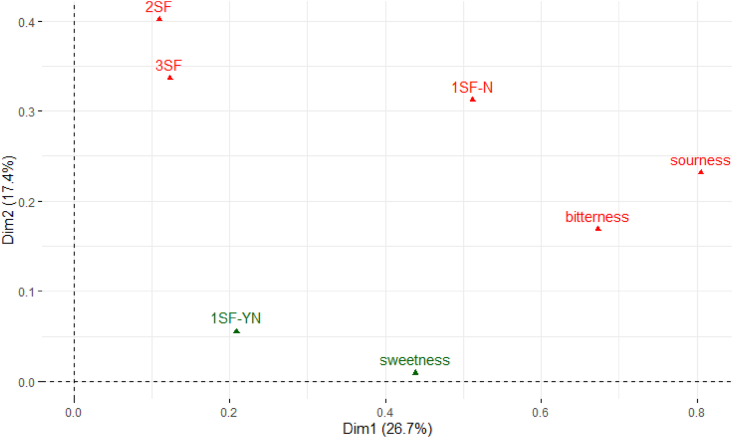
Overall MFA distribution of the PPM for all subjects with Dimension 1 contributing 26.7% and Dimension 2 contributing 17.4%.

**Fig. 3:**
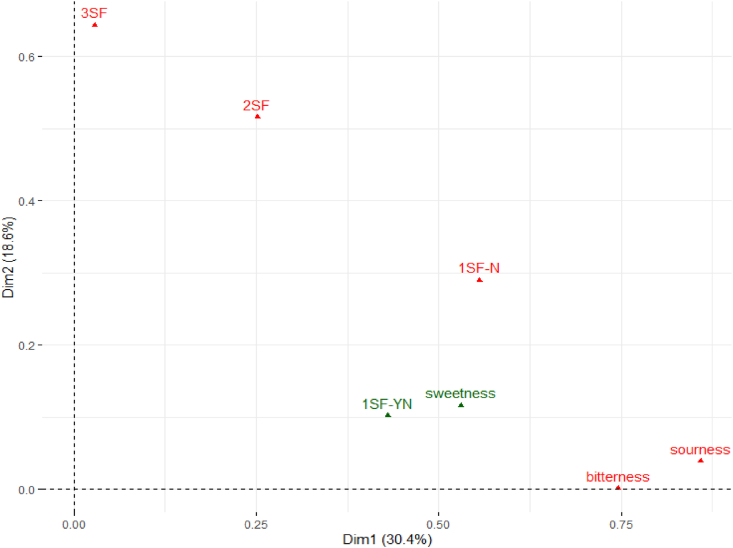
MFA distribution of the PPM for male subjects only with Dimension 1 contributing 30.4% and Dimension 2 contributing 18.6%.

**Fig. 4:**
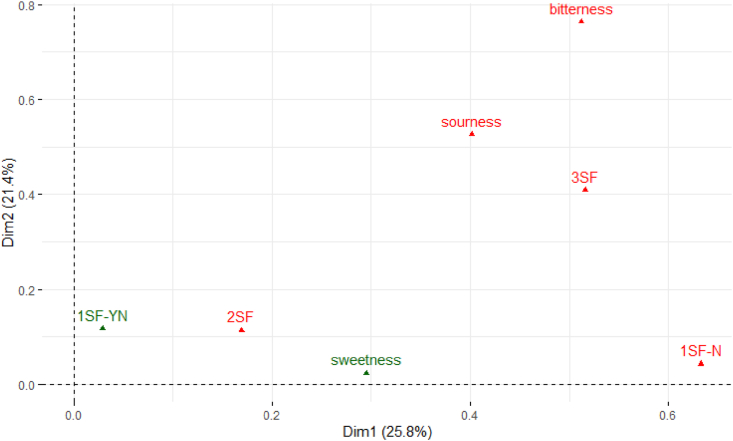
MFA distribution of the PPM for female subjects only with Dimension 1 contributing 25.8% and Dimension 2 contributing 21.4%.

For the sensory data, multiple factor analysis (MFA) was used to arrange the complex data, including quantitative and/or qualitative information on how the variables clustered together onto bi-plot maps (Pagès, 2005). The plots in Fig. 2, Fig. 3, and Fig. 4 represent the correlation between the seven solutions and dimensions on the bi-plot map. Within each bi-plot map, the position of each of the seven solutions shows how the three taste (such as sweet, sour, and bitter) solutions are associated with the different *makgeolli* samples. The overall MFA of the PPM data (Fig. 2) explains 44.1% (Dimension 1 = 26.7%, Dimension 2 = 17.4%) of the variation of the two dimensions. For the first dimension, sourness (citric acid), bitterness (quinine), and 1SF-N were identified as active groups which defines the distance between the variables in the bi-plot graph and contributed the most information. In comparison, three active groups (1SF-N, 2SF and 3SF) were identified and contributed the most information in dimension 2. Both 1SF-YN and sweetness were identified as supplementary data which have minimum influence in the overall MFA bi-plot graph. The male MFA data (Fig. 3) showed similar results compared to the overall MFA (Fig. 2), with the male MFA data explaining 49.0% (Dimension 1 = 30.4%, Dimension 2 = 18.6%) of variation of the two dimensions. In contrast, the female participants’ MFA (Fig. 4) showed 47.2% (Dimension 1 = 25.8%, Dimension 2 = 21.4%) of variation of the two dimensions on the bi-plot map. There is a consensus between the genders that bitterness, sourness, 1SF-N, 2SF, and 3SF are active groups in the MFA, while sweetness and 1SF-YN are both supplementary groups in this sensory experiment. However, the contribution of Dimension 1 and Dimension 2 differ slightly between male and female subjects. For male subjects, sourness (citric acid), bitterness (quinine), and 1SF-N contributes over 20% of the cutoff point in Dimension 1, while 3SF and 2SF contributes over 30% in Dimension 2. For female subjects (Fig. 3), 1SF-N, 3SF, and bitterness (quinine) are the three active groups for Dimension 1, while bitterness (contributing over 41.2%), sourness (citric acid), and 3SF contributes over 20% in Dimension 2.

**Fig. 5:**
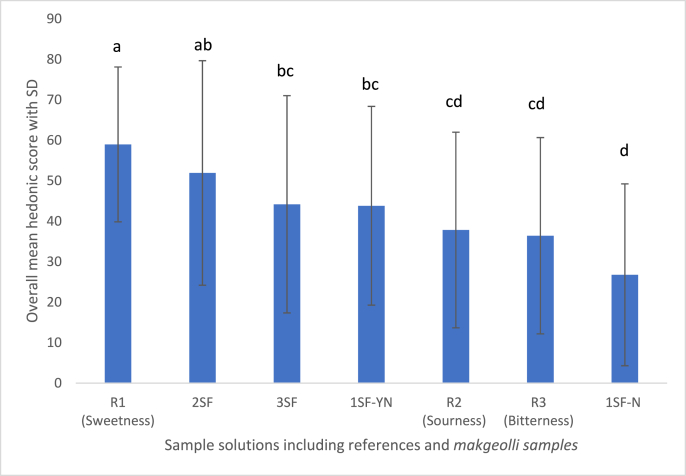
Overall mean hedonic score of samples tested in the PPM experiment with four types of makgeolli samples (1SF-YN, 1SF-N, 2SF and 3SF), sucrose, citric acid, and quinine solution (SD = standard deviation).

Analysis of variance showed a statistical significance in the overall mean hedonic rating among the reference solutions and the *makgeolli* samples (F value = 11.3, p = 9.84 x 10–12) as shown in Fig. 5. Sweetness was liked the most with an overall mean rating of 59.0 ± 19.1 mm on a 100 mm bi-polar scale, followed by the 2SF sample with an overall mean hedonic rating of 51.9 ± 27.7. Interestingly, the 2SF sample was perceived to be statistically different from the citric solution and was more closely associated with sweetness. The 3SF (44.2 ± 26.8 mm) and 1S-YN (43.8 ± 24.6 mm) samples had similar overall mean hedonic ratings in the PPM experiment and were statistically different from the sucrose and quinine solutions. Therefore, sourness is the best descriptor of both the 3SF and 1SF-YN samples. On the other hand, the 1SF-N sample had the lowest overall mean hedonic rating among the seven solutions in the PPM experiment (26.8 ± 22.5 mm). Bitterness had an overall mean hedonic score of 36.4 ± 24.2 and was statistically different to three *makgeolli* samples (2SF, 3SF, and 1SF-YN) but not to the 1SF-N sample.

In terms of gender differences between females and males, it was evident that female participants tended to score higher in the mean hedonic rating of all seven samples compared to males. However, female participants also had a higher standard deviation for most sample solutions. A statistically difference was observed for both female (F value = 6.13, p = 6.04 x 10-6) and male subjects (F value = 5.12, p = 6.29 x 10-5).

From the PPM experiment, increased fermentation time did not increase the subjects mean hedonic score. The 2SF sample had a longer fermentation time than the 1SF-YN and 1SF-N by two days but it was two days shorter than the 3SF sample. The two-stage fermentation process enhanced the sweetness of the *makgeolli* sample and participants tend to group the 2SF sample and the sucrose solution together as shown in the MFA bi-plot maps. In contrast, citric (sourness) and quinine (bitterness) solutions were perceived as undesirable traits. It was not surprising that participants rated both citric and quinine solutions relatively low, however, it was interesting to see that the 1SF-N had the lowest mean hedonic score overall. The low mean hedonic score for sample 1SF-N is likely to be attributed to the high concentration of nuruk used for the 1SF-N fermentation during the seven-day process.

The current study assessed the effects of New Zealand consumer perception of *makgeolli* in an environment outside of the laboratory setting. Researchers in this field of sensory science have noted that there is a variability of overall liking for food products being assessed at home (home use test) compared to the laboratory (central location test). However, other researchers have also noted that non-laboratory settings increased the encouragement of participants when sensory experiments are being conducted (Zandstra et al., 2020). Although this current research design was limited to observing the participants' behaviour in non-laboratory settings, this study may give researchers, such as in the wine industry, and brewing industries, insight into how New Zealand consumers are likely to perceive *makgeolli* in the natural environment. It has been historically argued by sensory scientists that central location tests can reduce random biases from participants, when variables such as the laboratory layout, food sample presentation, and information and experimental protocols remain constant (Boutrolle et al., 2005). However, the COVID-19 pandemic has changed the way many sensory studies are conducted and implemented during the period of 2020–2022 (Varela, 2022).

In this study, we have identified the key attribute that drove the overall liking of *makgeolli* is being closely associated with sweetness. The 2SF sample was rated the highest for overall liking by both males and females, and this can be explained by the high concentration of maltose and glucose content in this sample. This result is consistent with findings demonstrated in the United States where consumers preferred their Korean rice wine to be sweeter (Kwak et al., 2015). Thus, brewers and the wine industry might consider the 2SF *makgeolli* for the New Zealand market, as the sensory characteristics were the most preferred and liking is a necessary component for product success.

Both sourness and bitterness were the main attributes that drove participants to dislike certain *makgeolli* samples in this study, and in particular the 1SF-N sample. This sample had the highest levels of malic and citric acid which explains the strong association of sourness that participants perceived from the 1SF-N sample during the PPM experiment. According to Jeon et al. (2010) malic acid is considered an undesirable compound in wine products; however, this has not been previously documented or observed in the scientific literature for *makgeolli*. Although this is a preliminary finding, future work to overcome the limitations of this study such as the relatively small sample size and the structure of the PPM experiment warrants further investigation.. The high concentration of malic acid is likely to be one of the factors that caused participants to rate the 1SF-N sample relatively low in overall liking compared to the other three *makgeolli* samples. In terms of bitterness, high phenolic content tends to cause food products to be bitter and the 1SF-N *makgeolli* sample contained considerably high TPC values when compared to the other *makgeolli* samples in the PPM study. Also, although phenolic compounds in alcoholic beverages may sometimes be desirable, Lentz (2018) noted that spoilage or burnt characteristics may be perceived as undesirable by consumers.

The novelty of this study comes in two parts. First, this study uses a mixed methods approach where chemical analysis was employed to quantify the proximate composition and the concentration of the free amino acids, sugars, alcohol content, and antioxidant activity of the four different *makgeolli* processing methods. For the qualitative component, a sensory study using PPM and an A3 piece of paper was employed to categorially identify how the four *makgeolli* samples were perceived by participants according to sweetness, bitterness, and sourness. on an A3 piece of paper. From this study, researchers can observe that a high concentration of maltose and glucose is likely to increase the overall liking of the food product, specifically the 2SF sample in this study. A high concentration of malic acid is not desirable in *makgeolli* which was observed with a low overall liking for the 1SF-N sample.

Second, to the best of the researcher's knowledge, this is the first time that a projective method study has been completed and executed outside of the laboratory environment with food samples. This research demonstrates that projective methods can be implemented outside the laboratory with the guidance of the researchers. However, due to the multiple ways of conducting projective methods in sensory science such as using computer software (e.g., FIZZ and RedJade) or a pen and an A3/A2 sheet, researchers in future should compare the different experimental techniques of project methods outside of the laboratory setting to get comparable results.

## 4 Conclusion

From the chemical analysis, results showed that the 1SF-N *makgeolli* sample had the highest alcohol content, crude protein, antioxidant activity, total phenolic content, and total free amino acid concentration of the four samples. However, in sensory testing, the 1SF-N sample was rated the lowest overall liking out of the four *makgeolli* processing methods. This is likely to be attributed to the close association of sourness and bitterness by the participants from the PPM experiment.

Incorporating an additional fermentation step before the main fermentation was found to produce *makgeolli* with the highest-level of glucose and maltose content. Participants seem to prefer this *makgeolli* processing method (2SF). The 1SF-YN *makgeolli* sample had the lowest alcohol and total free amino acids content among the four processing methods tested. This is not surprising due to its low input of *nuruk* and yeast when producing 1SF-YN. Pyruvic, lactic, and acetic acid were the most dominant organic acids in all the *makgeolli* samples with concentrations above 1 g/L. The findings from this study will give a better understanding for rice wine brewers and researchers on how processing affects the chemical and sensory properties of *makgeolli* and how this influences consumer perceptions and overall liking. In terms of producing and launching more rice wine products in New Zealand, more research is needed to understand consumer attitudes towards purchasing rice wine for consumption and how branding, product packaging, and context of consumption might play a part in changing perceptions of the rice wine.

## CRediT authorship contribution statement

**Barry Wong:** Methodology, development for all stages of work, Writing – original draft, preparation of manuscript, Visualization, for all work, Investigation, for all work, Formal analysis. **Kevin Muchangi:** Assisting with, Investigation, Formal analysis. **Edward Quach:** Supervision, with, Formal analysis, and operations. **Tony Chen:** Supervision, and, Methodology, development for, Formal analysis. **Adrian Owens:** Conceptualization, and, Methodology, development for, Formal analysis. **Don Otter:** Writing – review & editing. **Megan Phillips:** Writing – review & editing. **Rothman Kam:** Overall all, Conceptualization, for the project, Writing – review & editing, Funding acquisition, Project administration.

## Declaration of competing interest

The authors declare that they have no known competing financial interests or personal relationships that could have appeared to influence the work reported in this paper.

## Data Availability

No data was used for the research described in the article.
