# Supplementary material for: Characterisation of Korean rice wine (makgeolli) prepared by different processing methods
Source: Curr Res Food Sci. 2022 Dec 27;6:100420. doi: 10.1016/j.crfs.2022.100420 (PMC9816670; doi:10.1016/j.crfs.2022.100420)
Supplement: Multimedia component 1 [file mmc1.docx]

# Supplementary information

## SI.1 A photo of the sensory test kit that was sent out to participants for the PPM sensory experiment.


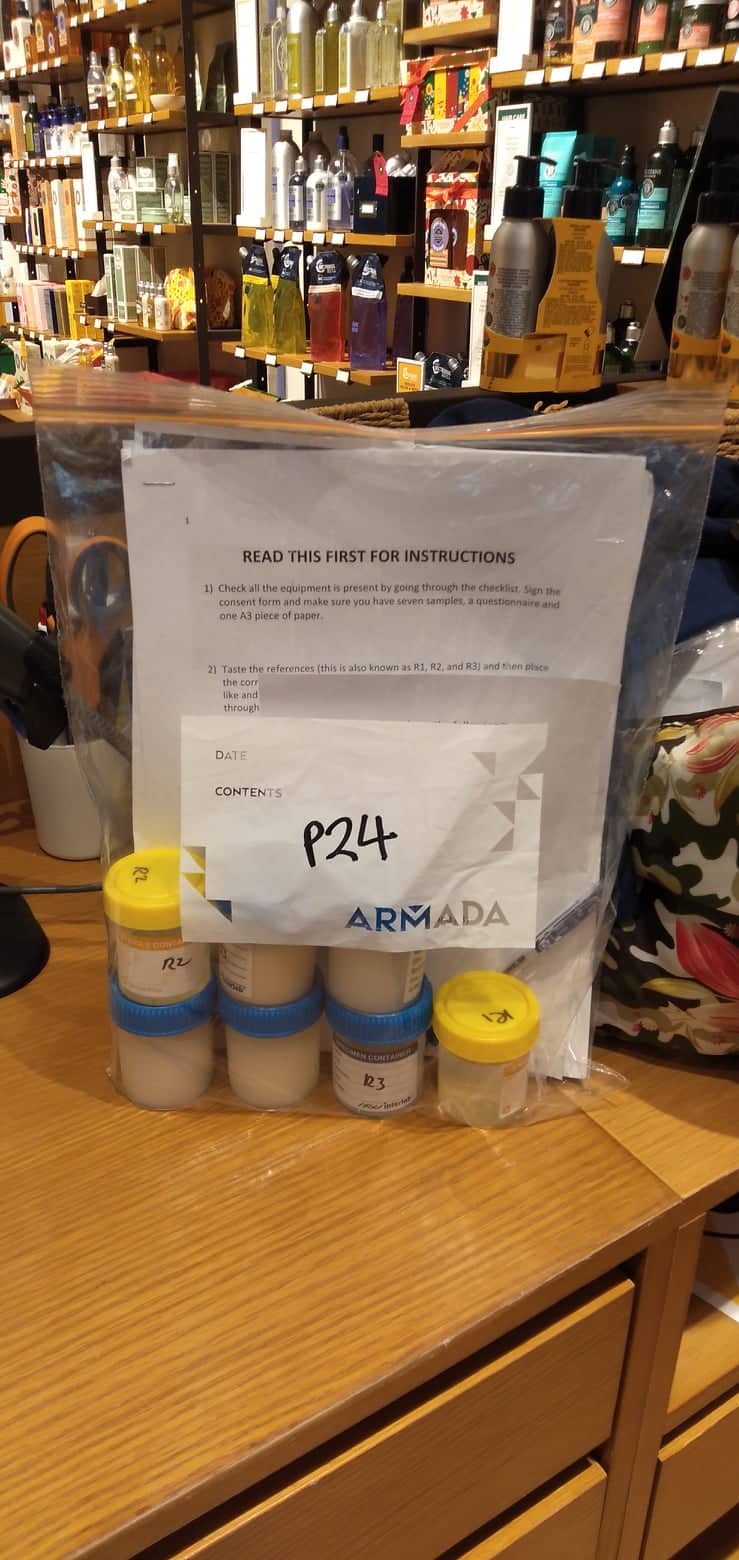


*SI.2 A photo demonstration of the location of the three poles, the colour is used to highlight the solution only and was not presented in the experiment.
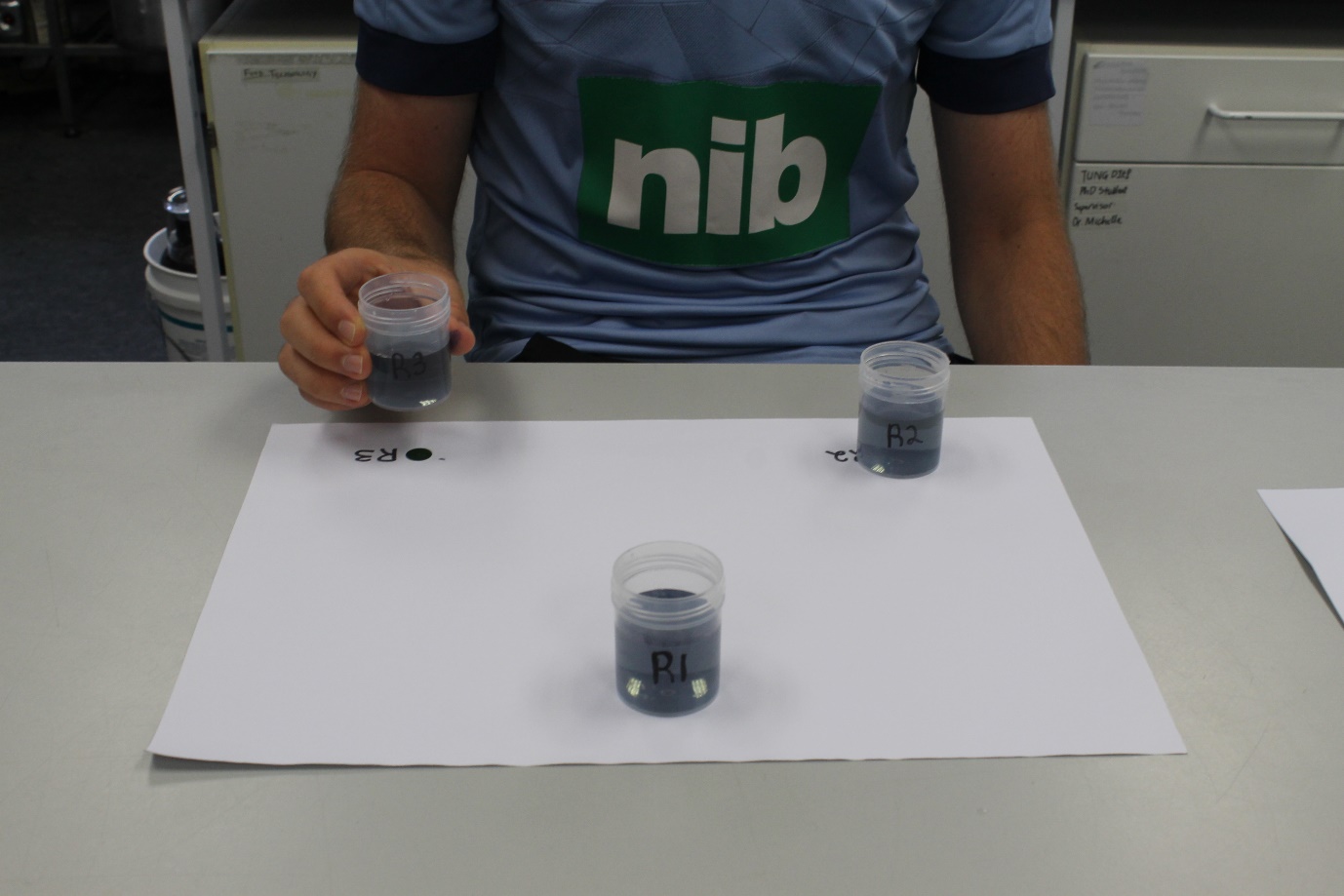
*
